# Severe finger necrosis in antisynthetase syndrome with positive anti‐OJ antibodies

**DOI:** 10.1002/ccr3.8990

**Published:** 2024-06-02

**Authors:** Yugo Horiuchi, Kenichi Hashimoto, Ryochi Yoshida, Akinori Sekizawa, Akatsuki Kubota, Meiko Maeda, Tatsushi Toda, Yuji Tanaka

**Affiliations:** ^1^ Department of General Medicine National Defense Medical College Tokorozawa Saitama Japan; ^2^ Division of Hematology and Rheumatology, Department of Internal Medicine National Defense Medical College Tokorozawa Saitama Japan; ^3^ Department of Neurology The University of Tokyo Tokyo Japan

**Keywords:** anti‐OJ antibodies, anti–synthetase antibody syndrome, immunoprecipitation, myositis, severe finger necrosis

## Abstract

**Key Clinical Message:**

In a patient with anti‐aminoacyl tRNA synthetase antibody and anti‐OJ antibody syndrome, interventions likes warming, prostaglandins, and antiplatelets failed. However, prednisolone pulse treatment rapidly halted disease progression. Patients with mild interstitial pneumonia, myositis, and extremity necrosis should be promptly considered for anti‐synthetase syndrome and receive immunosuppression after ruling out other causes.

**Abstract:**

Anti‐aminoacyl tRNA synthetase (ARS) autoantibodies are myositis‐specific, and patients who test positive for ARS and have common clinical features are usually diagnosed with antisynthetase antibody syndrome (antisynthetase syndrome). Anti‐ARS antibodies include histidyl‐tRNA synthetase‐1 (Jo‐1), anti‐threonyl (PL‐7), anti‐alanyl (PL‐12), anti‐glycyl (EJ), anti‐asparaginyl (KS), anti‐tyrosyl (Ha), and anti‐phenylalanyl (Zo) tRNA synthetases. Among these, anti–isoleucyl tRNA synthetase (OJ) autoantibodies are extremely rare, and patients with these are frequently complicated by interstitial pneumonia. We report the case of an older man with ARS antibody syndrome who tested positive for anti‐OJ and anti‐Sjögren's‐syndrome‐related antigen A (Ro‐52) antibodies. He had muscle weakness due to myositis and unparalleled rapid and severe finger necrosis. Pulsed prednisolone effectively treated the myositis symptoms and terminated the progression of finger necrosis.

## Graphical abstract

A purple color change was noticed in the second to fifth fingers of the left hand on admission (Fig. 1‐A). After admission, the color of the patient’s fingers changed from purple to black, indicating necrosis (Fig. 1B). After prednisolone pulse administration, a purple transition zone appeared in the necrotic area of the fingers, which subsequently ceased expanding (Fig. 1C). After pulse therapy, necrosis did not progress (Fig. 1D).

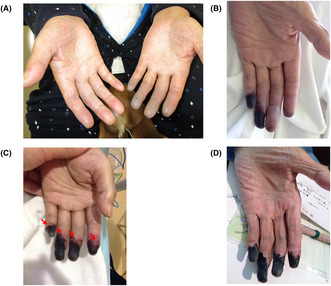

## 1 INTRODUCTION

Aminoacyl tRNA synthetases (ARSs) are a group of cytoplasmic enzymes that bind to transcribed RNA during protein synthesis, esterify amino acids to transcribed RNA, and function as catalysts for aminoacyl‐transcribed RNA.^1^ Patients who test positive for anti‐ARS antibodies present with various extramuscular symptoms, such as interstitial pneumonia, mechanic's hands, Raynaud phenomenon, polyarthritis, fever, and myositis. These symptoms share common clinical features, which, in 1992, led Targoff to term them “antisynthetase antibody syndrome.”^2^ Since Jo‐1 (histidyl‐tRNA synthetase: HisRS) antibody was discovered in 1980,^3^ eight ARS antibodies have been identified: histidyl‐tRNA synthetase‐1 (Jo‐1), anti‐threonyl (PL‐7), anti‐alanyl (PL‐12), anti‐glycyl (EJ), anti‐isoleucyl (OJ) anti‐asparaginyl (KS), anti‐phenylalanyl (Zo), and anti‐tyrosyl (Ha) tRNA synthetase.^4^, ^5^, ^6^ Among these antibodies, anti‐OJ is the least prevalent (3.1%).^7^ Anti‐OJ‐positive anti‐ARS syndrome is extremely rare and is usually accompanied by interstitial pneumonia.^8^ Anti‐OJ and anti‐Sjögren's‐syndrome‐related antigen A (Ro‐52) antibodies are occasionally detected in patients with dermatomyositis and are a poor prognostic factor when associated with interstitial pneumonia.^9^ However, we encountered a patient with OJ‐and Ro‐52 positive anti‐ARS syndrome without interstitial pneumonia and with severe phalanx necrosis due to the Raynaud phenomenon. The activity of the disease could be suppressed with steroid pulse therapy. We believe that this is an atypical clinical presentation that deserves acknowledgment by means of this case report. Additionally, we provide a review of the literature on the topic.

## 2 CASE HISTORY EXAMINATION

The patient was an 80‐year‐old Japanese man. During the month before admission, he experienced progressive dysphagia and muscle weakness in the lower extremities. Because of difficulty with walking, he sought medical attention at the hospital. He had a 60‐year history of smoking 10 cigarettes per day and visited the hospital infrequently; moreover, he was not currently on any medication. The patient had no history of the Raynaud phenomenon.

**FIGURE 1:**
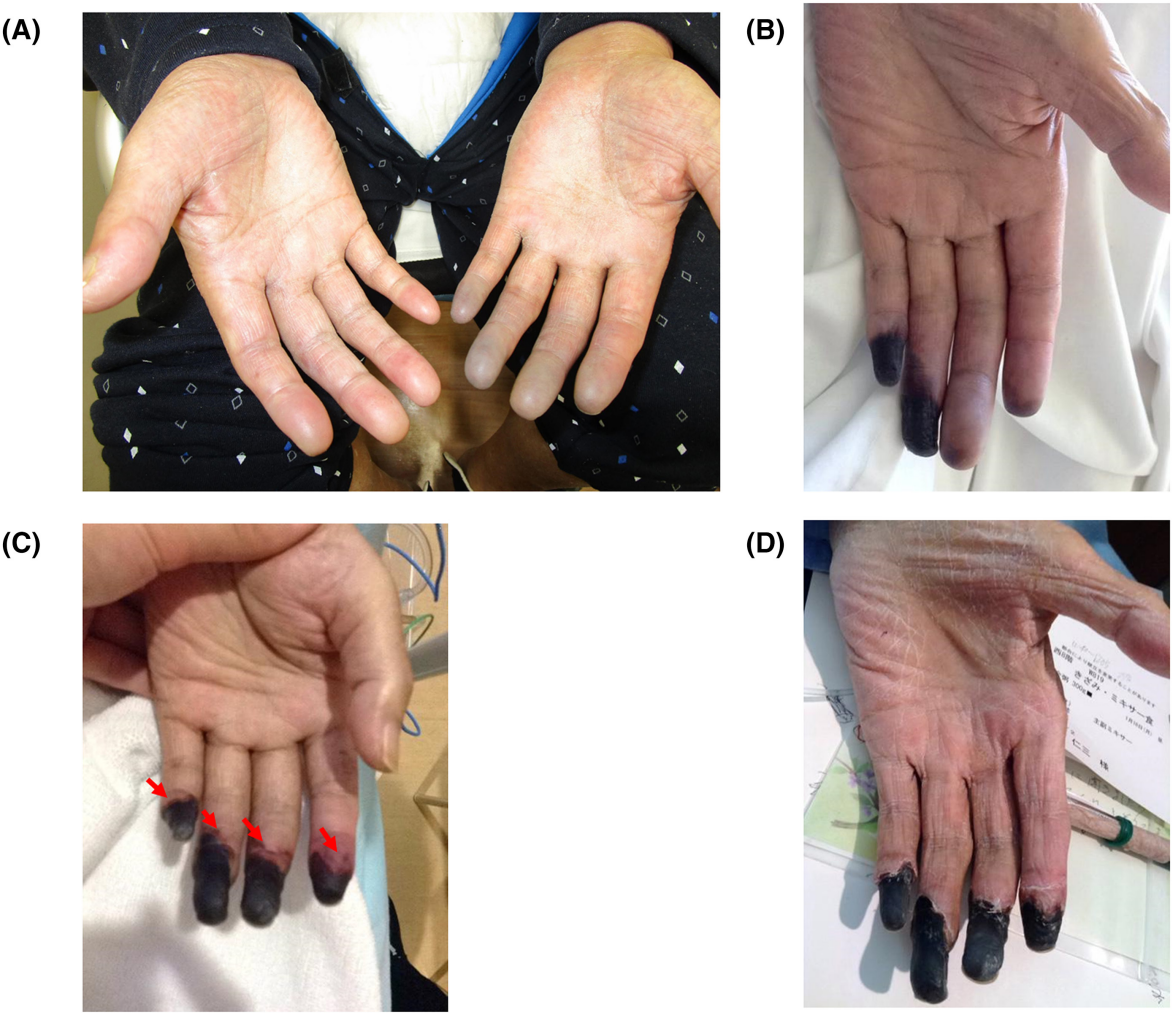
Imaging findings of affected fingers. Hospitalization days 1 (A), 3 (B), 10 (C) after the first prednisolone pulse and 70 (D) at discharge. The red arrow (D) shows a purple transition zone between the black necrotic area and normal skin.

On admission, the patient's temperature was 37.3°C, and no other abnormalities were detected in his vital signs. Indurated edema was observed in both lower legs, and a purple color change was noticed in the second to fifth fingers of the left hand (Figure 1A). Although muscle atrophy was not evident because of edematous changes, the patient experienced generalized muscle pain in the bilateral thighs. The strength in the upper extremities was assessed as a Muscle Strength Testing (MMT) score of 4, while the iliopsoas and quadriceps muscles were graded as an MMT score of 2.

## 3 DIFFERENTIAL DIAGNOSIS, INVESTIGATIONS, AND TREATMENT

Table 1 shows laboratory data on admission. Blood test findings revealed elevated levels of muscle enzymes (creatine kinase [CK], 7930 U/L, and C‐reactive protein [CRP], 2.0 mg/dL), an erythrocyte sedimentation rate (ESR) of 16 mm/h, and an elevated inflammatory response. Results were negative for antinuclear antibodies, rheumatoid factor (RF), myeloperoxidase‐anti‐neutrophil cytoplasmic antibodies (MPO‐ANCA), proteinase‐3‐anti‐neutrophil cytoplasmic antibodies (PR3‐ANCA), anti‐Scl‐70, U1RNP, centromere, Jo‐1, PL‐12, anti‐melanoma differentiation‐associated gene‐5 (MDA5), anti‐transcriptional intermediary factor 1‐γ (TIF1‐γ), as well as MI‐2, Ku, PM‐SCL75, PM‐SCL100, SRP, PL‐7, EJ, and OJ. However, Ro‐52 (EUROLINE Myositis Profile 3; EUROIMMUN Medizinische Labordiagnostika AG, Lubeck, Germany) demonstrated a strong positive result (3+). During the patient's hospitalization, further testing using immunoprecipitation was conducted at another institute's laboratory, and the patient was strongly positive for anti‐OJ antibodies.

**TABLE 1:** Laboratory data on admission.

Laboratory data				
	Test result	Reference value	Test result	
Peripheral blood			Myositis panel (EUROLINE)	
WBC	7600	3300–8600 μL	MI‐2 Autoantibodies	(±)
Hemoglobin	12.1	13.7–16.8 g/dL	Ku Autoantibodies	(−)
Platelets	18.3 × 104	15.0–40.0 × 104 μL	PM‐SCL100	(−)
			PM‐SCL75	(−)
Blood chemistry			SRP Autoantibodies	(−)
BUN	25	8–20.0 mg/dL	PL‐7 Autoantibodies	(−)
Creatinine	0.99	0.61–1.13 mg/dL	OJ Autoantibodies	(−)
eGFR	56	0 mL/min/1.73m^2^	EJ Autoantibodies	(−)
AST	419	5–30.0 U/L	Anti‐MDA‐5	(−)
ALT	176	5–35 U/L	Anti‐TIF1‐γ	(−)
CK	7390	<16 U/L	Jo‐1 Autoantibodies	(±)
ESR	16	45298 mm/1h	PL‐12 Autoantibodies	(±)
CRP	2	<0.3 mg/dL	RO‐52 Autoantibodies	(+++)
Immunoserology			(Immunoprecipitation)	
Anti‐nuclear antibodies (ANA)	<40	<40	OJ Autoantibodies	(+)
Rheumatoid factor (RF)	16	≦20		
Anti‐CCP antibody	<0.6	<4.5		
C3	52	65–135		
C4	24	13–35		
Jo‐1 antibody	(−)			
PR3‐ANCA	<1.0	<1.0		
MPO‐ANCA	<1.0	<1.0		
Scleroderma‐(ScI70) antibody	(−)			
Anti‐dsDNA	(−)			
Anti‐SM	(−)			
Anti‐anti‐Sjögren syndrome related Ag A (SS‐A)	(−)			
Anti‐anti‐Sjögren syndrome related Ag B (SS‐B)	(−)			
Anti‐RNP antibody	(−)			
Anticardiolipin antibodies (ACA)	(−)			
Anti‐β2‐glycoprotein I antibodies (Anti‐β2‐GPI)	(−)			

Abbreviations: ALT, alanine aminotransferase; AST, asparate transaminase; BUN, blood urea nitrogen; CCP, Cyclic citrullinated peptide; CK, creatine kinase; CRP, C reactive protein; eGFR, estimated glomerular filtration rate; ESR, Erythrocyte sedimentation rate; MDA, Melanoma‐derived‐antigen‐5; MPO‐ANCA, Myeloperoxidase‐Anti‐neutrophil cytoplasmic antibodies; PR3‐ANCA, proteinase 3‐Anti‐neutrophil cytoplasmic antibodies; TIFI1, Anti‐transcription intermediary factor 1; WBC, White blood cell.

**FIGURE 2:**
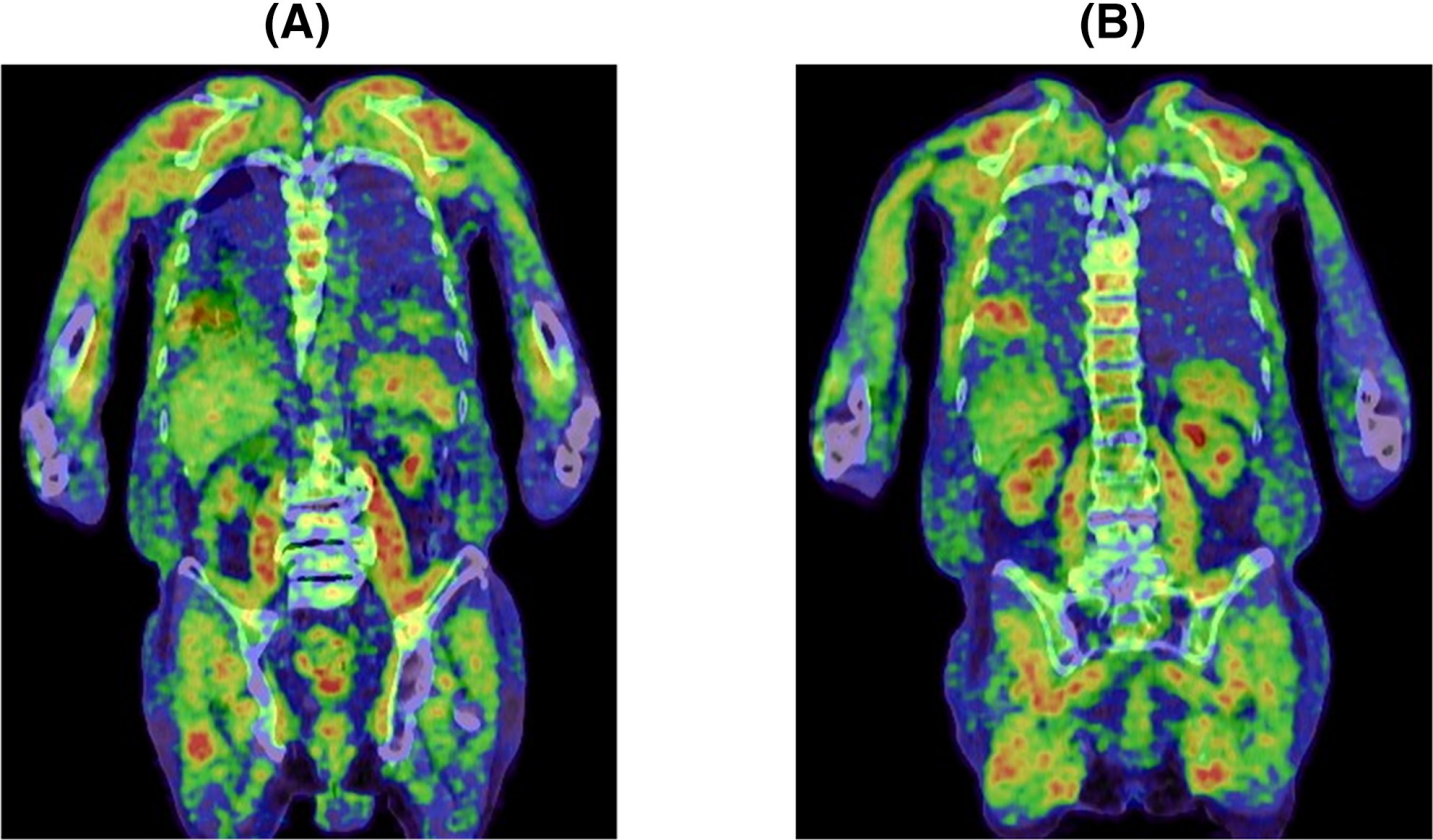
PET‐CT imaging findings on day 1 of hospitalization. Widespread accumulation centered on (A) soft shoulder tissues and on (B) quadriceps muscle. Blood glucose levels are normal. PET‐CT, positron emission tomography‐computed tomography.

**FIGURE 3:**
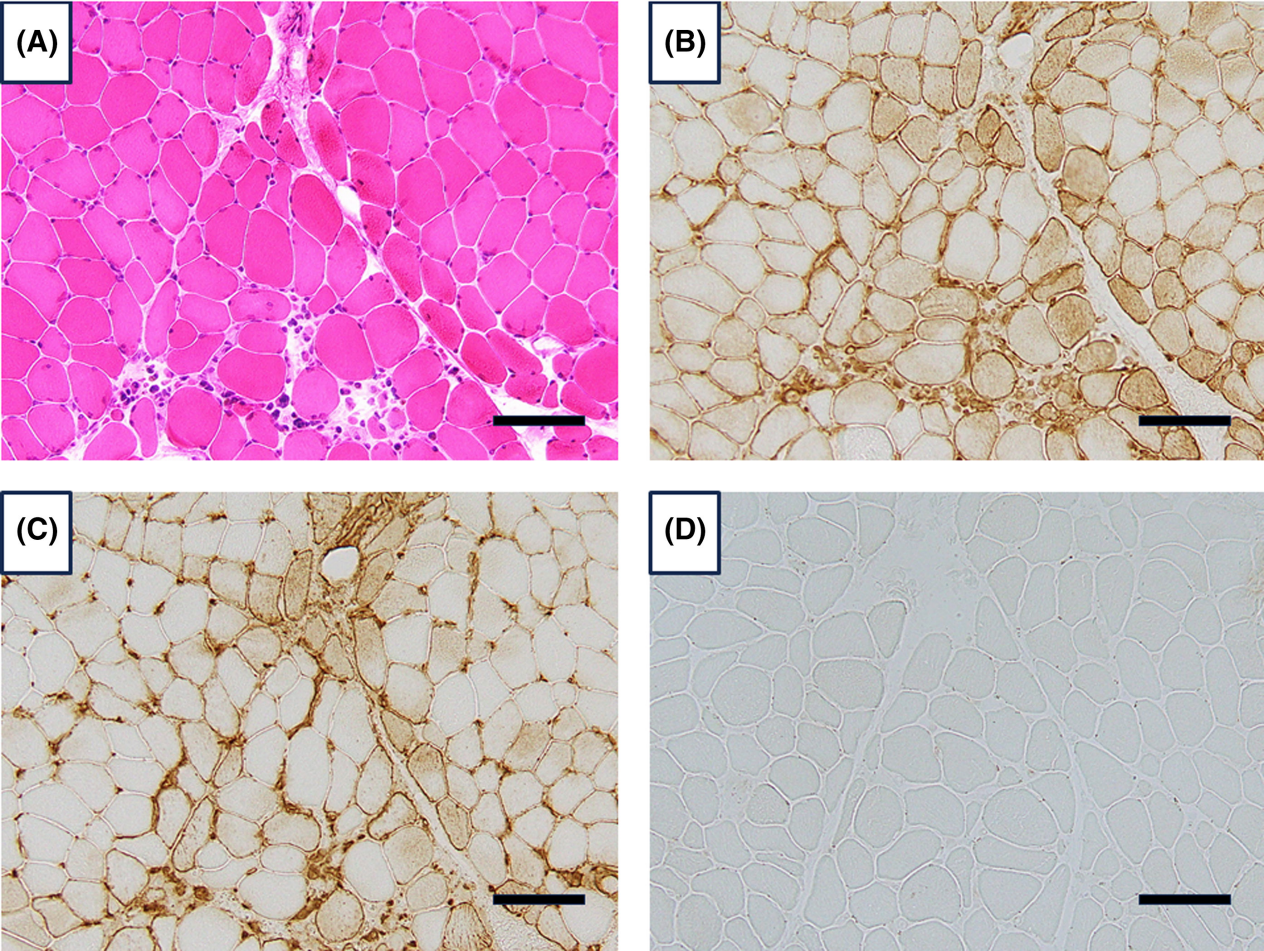
Myopathological features of the patient. Hematoxylin & eosin staining (A) shows mild fiber size variation, mild endomysial fibrosis, and a few necrotic fibers. Immunostaining with the anti‐HLA‐ABC antibody (B) reveals positivity of most fibers, with perifascicular areas appearing darker. Immunostaining with the anti‐HLA‐DR antibody (C) demonstrates positivity predominantly in perifascicular areas. Immunostaining with the anti‐MxA antibody (D) does not show positivity in myofibers. Bars indicate 100 μm.

Contrast‐enhanced magnetic resonance imaging (MRI) revealed a contrast effect in muscle, The MRI findings indicated that the quadriceps femoris muscle exhibited no high signal on T1W1 but a high signal on T2W1 and STAIR, consistent with myositis. The positron emission tomography‐computed tomography also showed extensive soft tissue accumulation despite normal blood glucose levels (Figure 2A,B). A muscle biopsy was performed on the left biceps brachii muscle. On histochemistry, mild fiber size variation, mild endomysial fibrosis, and a few necrotic fibers were observed (Figure 3A–D). Inflammatory cell infiltrates were minimal. On immunohistochemistry, HLA‐ABC and HLA‐DR were positive on most fibers, and especially perifascicular areas were stained darker, suggesting a perifascicular pathology. Immunostaining with anti–myxovirus resistance protein A (MxA) antibody showed no positivity on muscle fibers. These findings supported a diagnosis of anti‐ARS antibody syndrome. Chest computed tomography (CT) upon admission indicated chronic obstructive pulmonary disease and mild interstitial pneumonia; however, no malignancy was detected. MRI of the head showed no abnormality.

After admission, the color of the patient's fingers changed from purple to black, indicating necrosis (Figure 1B). Contrast‐enhanced CT images showed no signs of vascular stenosis or obstruction. Despite treatment with intravenous heparin sodium (10,000 U/day), prostaglandins (limaprost alfadex 30 μg/day), tocopherol nicotinate 600 mg/day, and warming agents for a week, the progression of the finger necrosis did not stop.

Due to the severe finger necrosis, anti‐phospholipid syndrome and cryoglobulinemia should be considered in the differential diagnosis. However, because lupus anticoagulant, anti‐cardiolipin antibody, anti‐cardiolipin β2 glycoprotein I complex antibody, complement (C3 and C4), and cryoglobulin were all negative, anti‐phospholipid syndrome and cryoglobulinemia were excluded from the differential. Additionally, the patient exhibited a significantly elevated high serum CK level, which warranted continued fluid replacement therapy to protect renal function.

On day 10 of hospitalization, the patient's respiratory status deteriorated, necessitating ventilator management (requiring the administration of 10 min of masked oxygen). The possibility of exacerbation of interstitial pneumonia, we opted for methylprednisolone pulse treatment (methylprednisolone pulse of 1000 mg for 3 days and post‐therapy prednisolone [PSL]; 60 mg [1 g/kg]). Close examination revealed no exacerbation of interstitial pneumonia or changes in Krebs von den Lungen 6 (KL‐6; 216 U/mL) or surfactant protein‐D (SP‐D; 65 ng/mL). Imaging revealed a mildly exacerbated gland glass shadow without elevated KL‐6 or SP‐D. He also had a history of chronic obstructive pulmonary disease as a cause of worsening respiratory status, which was thought to be due to CO_2_narcosis. Following the initiation of treatment, his respiratory condition rapidly improved, and he was weaned off the ventilator within approximately 10 days. This was attributed to improved carbon dioxide (CO_2_) narcosis. After the first day of PSL pulse administration, a purple transition zone appeared in the necrotic area of the fingers, which subsequently ceased expanding (Figure 1C). Although we considered immunosuppressive therapy, we did not use it because the methylprednisolone pulse stopped the progression of finger necrosis, the patient's general condition was stable, and the prednisolone taper was progressing well. Moreover, we took prophylactic measures by using sulfamethoxazole‐trimethoprim, proton pump inhibitors, and alendronate, while paying attention to blood glucose, to prevent side effects. The patient experienced repeated episodes of aspiration pneumonia owing to dysphagia; however, over time, both swallowing ability and lower limb muscle strength gradually improved. On day 40 of hospitalization, the MMT score of the lower limbs recovered to 3–4, and CK levels were normal. Additionally, the CRP level and ESR had substantially decreased. Furthermore, the progression of finger necrosis had ceased. By day 70 of hospitalization, all MMT scores had improved to 5, indicating complete recovery of muscle strength. Swallowing function had also improved to the state before the disease onset, leading to the patient's discharge from the hospital. Although the necrotic areas of the fingers became narrower and drier after pulse therapy, necrosis did not progress (Figure 1D).

## 4 OUTCOME AND FOLLOWUP

Following discharge, the patient was transferred to a rehabilitation hospital for further care. After 2 months of rehabilitation, the patient was discharged from the rehabilitation hospital and did not experience any recurrence of symptoms. Furthermore, there was no progression of necrosis in the fingers following discharge from our hospital. A team conference was conducted involving nurses, collagen disease physicians, a plastic surgeon, an orthopedic surgeon, and a family member to discuss the possibility of amputating the necrotic fingers. Considering that the patient was receiving PSL and that future use of immunosuppressive drugs was contemplated, it was decided not to proceed with amputation because of the associated risk of infection. Instead, the patient's progress would continue to be monitored.

## 5 DISCUSSION

We diagnosed an older patient with rapidly progressive finger necrosis and late‐onset ARS syndrome who tested positive for anti‐OJ and Ro‐52 antibodies. The occurrence of ARS syndrome with positive anti‐OJ antibodies is extremely rare, and reports of clinical manifestations of severe necrosis of the hands without significant interstitial pneumonia are limited.^8^ Additionally, the commonly used line immunoassay (LIA) method for measuring anti‐OJ antibodies yielded false negative results in this case, highlighting the effectiveness of immunoprecipitation.

Ge et al.^7^ reported that only 10 (3.1%) of 320 patients with anti‐ARS antibodies had anti‐OJ antibodies. Among them, 90% had interstitial pneumonia, and 40% presented with myositis, mechanic's hands, and arthritis. Most publications describing patients with anti‐OJ antibodies have primarily focused on interstitial pneumonia,^8^ with only one report mentioning myositis^9^ and none describing skin changes with necrosis. Our patient experienced mild interstitial pneumonia, while muscle weakness and necrosis of the fingers were prominent. Although the patient remained independent in activities of daily living, he faced difficulties in swallowing and lost the ability to walk after just 1 month. Finger necrosis was notably absent upon admission but progressed rapidly and intensely within 3 days.

On admission, anti‐Ro‐52 was strongly positive. Notably, anti‐Ro‐52 positive ARS syndrome is associated with a poor prognosis when it occurs alongside concomitant interstitial pneumonia.^10^ Anti‐Ro‐52 antibodies are found in up to 37% of patients with myositis, and this often correlates with anti‐Jo‐1 reactivity.^11^ This, together with the clinical presentation, indicated myositis involvement. However, anti‐Jo‐1 and anti‐ARS antibodies were all negative. Few reports have described finger necrosis due to myositis, and we considered the possibility of a paraneoplastic acral vascular syndrome.^12^ Consequently, we requested a test for anti‐U5snRNP antibody, which was negative. Nevertheless, we retested the anti‐OJ antibody by immunoprecipitation, which was positive. In the 10 cases reported by Ge et al.^7^ 6/10 exhibited cytoplasmic speckled patterns, the most common type, 3/10 showed nuclear‐speckled patterns, the second most common, and 1/10 was ANA negative. Although there are other scattered reports of ANA‐negative cases by Sekiya et al.^13^ such cases are considered rare in previous literature.

We considered initiating PSL at a dose of 1 mg/kg for the patient while awaiting the examination results. However, because of the sudden deterioration of the patient's respiratory status (necessitating the administration of 10 min of masked oxygen) and the possibility of exacerbation of interstitial pneumonia, we opted for methylprednisolone pulse treatment. Imaging revealed a mildly exacerbated interstitial shadow without elevated KL‐6 or SP‐D. Bartella et al.^14^ reported that nonspecific interstitial pneumonia (NSIP) is the most common presentation in anti‐ARS syndrome, accounting for 27% of cases. In the present case, the chest CT image at the time of the sudden change showed frosted shadows centered on the dorsal area, which is not a typical presentation of NSIP in patients with anti‐ARS syndrome. However, the disappearance of the shadows in about 10 days and the absence of elevations in KL‐6 and SP‐D led us to believe that the main cause of acute respiratory failure was aspiration pneumonia with mild interstitial lung disease in the base. He also had a history of chronic obstructive pulmonary disease as a cause of worsening respiratory status, which was thought to be due to CO_2_ narcosis. After administering the methylprednisolone pulse, the expanding necrotic lesion, which had shown no signs of prior improvement, exhibited a purple transition zone at the border between the normal skin and the black necrotic area (Figure 1C). Subsequently, the expansion of necrosis halted. The pathogenesis of finger necrosis in anti‐ARS syndrome has been documented. For example, Chan et al.^15^ reported a PL‐7(+) case where conventional angiography revealed occlusion of the second to fourth fingers. Suma et al.^16^ reported Jo‐1(+) anti‐ARS antibody (+) mechanic's hand, with peripheral angiography revealing occlusion of both the ulnar and radial arteries. Linear hemorrhage of fingernails accompanied by digital ischemia or digital ulcer in anti‐ARS syndrome has also been reported.^15^, ^16^, ^17^ In our case, no obvious stenosis or occlusion was observed on contrast‐enhanced CT angiography, and there was no linear hemorrhage of the nails. However, we observed extreme necrosis, which was unprecedented in our experience. Nonetheless, the presence of necrosis would typically suggest ischemia caused by blood flow obstruction in the vessel, which may be a limitation of CT angiography. Furthermore, because of the patient's intubation and poor general condition, angiography was not performed. Although the patient had no history of the Raynaud phenomenon, spasms of the peripheral arteries in the fingers might have contributed to the significant necrosis. However, it is unlikely that spasm alone would have caused this degree of finger necrosis. Although speculative, vasculitis at the level of the finger microvasculature may have also played a role. Involvement of finger microvessel vasculitis in ARS syndrome‐related finger necrosis has been reported.^15^, ^16^ Therefore, the combined effect of spasm and vasculitis at the microvascular level in this case cannot be dismissed. This case merits reporting for several reasons: the rarity of digital necrosis in patients positive for anti‐OJ antibodies, and notably, the significant improvement in muscle strength following PSL administration, which underscores the efficacy of PSL pulses in these patients.

The limited number of reports may be attributed to the rarity of this condition and challenges in testing. Anti‐OJ antibodies are difficult to detect among all myositis‐specific antibodies. Unlike other tRNA synthetase targets of autoantibodies, including Jo‐1, PL‐7, PL‐12, KS, EJ, and Zo, OJ antibodies are complex high‐molecular‐weight proteins with several subunits, posing challenges for antigen selection.^18^ The LIA method has lower sensitivity in detecting anti‐OJ antibodies.^19^, ^20^, ^21^ However, it is still widely applied in clinical practice owing to the lack of alternative methods and its ease of use, despite the superior diagnostic performance of advanced immunoprecipitation methods.^22^, ^23^ In this case, although the patient initially tested negative for anti‐OJ antibodies using the LIA method, he tested positive based on the immunoprecipitation method. The single enzyme‐linked immunosorbent assay kit commonly used for the anti‐ARS antibody assay no longer detects anti‐Zo and anti‐Ha antibodies, as they have only been reported in isolated cases.

If clinicians suspect the presence of anti‐OJ antibodies and myositis and finger necrosis are more prominent than symptoms of interstitial pneumonia, a diagnosis should be confirmed through immunoprecipitation.

## 6 CONCLUSION

We described the case of an older patient with late‐onset ARS syndrome who tested positive for anti‐OJ and anti‐Ro‐52 antibodies. Interstitial pneumonia is an established complication of anti‐ARS antibody syndrome, particularly when accompanied by positive anti‐OJ antibodies. However, it can also result in rapid necrosis of the fingers without interstitial pneumonia. Prednisolone pulse therapy was remarkably effective, leading to the rapid cessation of disease progression. Detecting anti‐OJ antibodies by immunoprecipitation is potentially more reliable than the LIA method. Information on the clinical course of this atypical ARS syndrome and treatment could contribute to the daily practice of rheumatology for clinicians.

## AUTHOR CONTRIBUTIONS

**Akatsuki Kubota:** Data curation; writing – review and editing. **Kenichi Hashimoto:** Conceptualization; data curation; project administration; supervision; writing – review and editing. **Meiko Maeda:** Data curation; writing – review and editing. **Ryochi Yoshida:** Supervision; writing – review and editing. **Akinori Sekizawa:** Data curation; writing – review and editing. **Tatsushi Toda:** Validation; writing – review and editing. **Yuji Tanaka:** Supervision; writing – review and editing. **Yugo Horiuchi:** Conceptualization; data curation; project administration; writing – original draft.

## FUNDING INFORMATION

None.

## CONFLICT OF INTEREST STATEMENT

Not applicable.

## ETHICS STATEMENT

The study was approved by the Competent Authorities and Ethics Committees of National Defense Medical College (approval no. 4543). Written informed consent was obtained from the patient.

## CONSENT

Written informed consent was obtained from the patient to publish this report in accordance with the journal's patient consent policy.

## Data Availability

The data that support the findings of this study are available on request from the corresponding author. The data are not publicly available due to privacy or ethical restrictions.
